# Factor Structures of General Health Questionnaire-12 Within the Number of Kins Among the Rural Residents in China

**DOI:** 10.3389/fpsyg.2019.01774

**Published:** 2019-08-02

**Authors:** Ming Guan, Bingxue Han

**Affiliations:** ^1^Family Issues Center, Xuchang University, Xuchang, China; ^2^School of Business, Xuchang University, Xuchang, China; ^3^College of Urban and Rural Planning and Gardening, Xuchang University, Xuchang, China

**Keywords:** factor structure, GHQ-12, number of kins, mental health, rural residents

## Abstract

The 12-item general health questionnaire (GHQ-12) has been extensively used with Chinese population. Yet, it has not been used from a national representative survey in rural China. The aim of this study was to examine how number of kins associated with the factor structures of the CHQ-12 among the rural residents in China. Data were obtained from the 2009 rural-to-urban migrants survey (RUMiC). Exploratory factor analysis (EFA) (principal component analysis with varimax rotation) was performed to identify factor structures of GHQ-12 regarding number of siblings, age ranking, and number of children. To investigate the reliability of the questionnaire, Cronbach’s alpha was used. Internal consistency was assessed by confirmatory factor analysis (CFA). In all, 32171 rural residents in China from 2009 RUMiC participated in the study. The mean age of the respondents was 37.03 (*SD* = 19.21) years. The psychometric properties and factor structures of the GHQ-12 used were described. All of the fit indices in CFA models were satisfactory. The two-factor and three-factor structures gathered the satisfactory fit indexes in the part of 2009 subsamples. The rural version of the GHQ-12 was reliable measures of psychological distress among the rural residents in China with respect to number of kins. The two-and three-factor structures derived from the present sample, with good model fit in the CFA analysis, which suggested that two-and three-factor solution could be used to assess mental health of rural residents in rural China.

## Introduction

The most prevalent mental disorders have been reported in mainland China. The prevalence rates of anxiety disorders (e.g., Guo et al., 2016), schizophrenia (e.g., Chan et al., 2015), poor mental health status among older population (Wang et al., 2016), and older adults loneliness (e.g., Zhong et al., 2018) were particularly high in modern China. In rural China, prevalence of reproductive depression (Cao et al., 2015), elderly suicide rate (e.g., Zhong et al., 2016), adolescents aggression (Huang et al., 2017), mental health problems among left-behind children (Tang et al., 2018; Wang J. et al., 2019) and spouses (Nikoloski et al., 2019), students anxiety (e.g., Liu H. et al., 2018), self-stigma (Ran et al., 2018), general depression (Qiu et al., 2018), and hopelessness among lower-class rural residents (Zhang et al., 2019) were high. Mental disorders were significantly associated with increased risk of suicide attempt in rural China (e.g., Sun et al., 2015; Liu et al., 2017; Liu B.P. et al., 2018). A cross-sectional survey concluded family function rather than family structure had key impact on mental health among rural residents (e.g., Cheng et al., 2017). A psychiatry study in rural China showed increased risk of suicide attempt was associated with family conflicts (Liu et al., 2019). Thus, it is necessary to explore the relationship between number of kins and prevalence of mental disorders measured in rural China.

Several studies have addressed the influence of the number of kins on people’s psychopathology in the cases of mental disorders in different developmental stages. For example, a systematic review reported that sibling influences depended on siblings’ frequent and emotional intense interactions and siblings’ role in large family system dynamics (McHale et al., 2012). Considering age ranking, recent adolescence literature has confirmed the psychological interactions between older siblings and younger siblings during adolescence with increasing age (e.g., Eckstein et al., 2018; Wang M.T. et al., 2019). These studies reflected the psychological relationships between adolescents rather than adult siblings. Regarding number of children, another study suggested that the interdependence between parents and children persisted once children reached adulthood (Lee et al., 2016). Since part of elders in late-life depended on family’s support, childless elders in rural China often faced high risk of depression (Guo, 2014). Another gerontology study observed contacts with adult children might depress older fathers in China (e.g., Wu and Fokkema, 2018). Thus, number of kins may be an important indicator to reflect the prevalence of mental disorders measured by 12-item general health questionnaire (GHQ-12) in rural China.

The GHQ-12 designed by Goldberg (1972) is often used to screen for non-specific psychiatric morbidity. The GHQ-12 is a widely used screening instrument for detecting depression (Ozdemir and Rezaki, 2007), mental illness (Donath, 2001; Hu et al., 2007), anxiety/depression (e.g., Uras et al., 2012), and depressive disorder (e.g., Doyle et al., 2012; Lundin et al., 2016) in a variety of settings across countries. It costs only a few minutes to complete and score (Picardi et al., 2004). Regarding life stages, adolescents were interpreted by the GHQ-12 in a similar manner to adults (French and Tait, 2004). Considering national difference, German (Schrnitz et al., 1999), Iranian (Montazeri et al., 2003), Spanish (López-Castedo and Fernández, 2005), Portuguese (Laranjeira, 2008), and bilingual Chinese/Italian (Barbato et al., 2009) version of GHQ-12 as a whole were reliable questionnaires for measuring psychological well-being (Tait et al., 2003). GHQ-12 was both culturally valid and psychometrically sound in the Chinese rural context (Lee et al., 2006). Chinese version for professional groups (Liang et al., 2016), the general population (Shek, 1987), clinical groups (Gao et al., 2004), and Chinese women (Ip and Martin, 2006) had high internal consistency. The eight-item two-factor model was the best-fit model in Chinese adolescents (Li et al., 2009). Although certain notable discrepancies were observed at the item level, the English and Chinese versions of the GHQ were comparable at the scale level (Chan, 1985). Prior studies showed factor structure of GHQ-12 was influenced by socioeconomic factors. For example, factor structure of GHQ-12 might depend on national difference (Salama-Younes et al., 2009) and gender difference (Doi and Minowa, 2003). In fact, GHQ-12 was used to measure individual mental health. It is the common sense that factor structure of GHQ-12 possibly would be influenced by respondents’ family characteristics. But, till now, little results reported the relevant research.

Due to son preference, urban family size often was smaller than rural family size in China. This study aimed to evaluate how number of kins influenced the factorial structure of the Chinese version of GHQ-12 among the rural residents. A large sample from a publicly available survey dataset was adopted here. Within the context, factor structure of the Chinese version of GHQ-12 would be identified with descriptive, exploratory, and confirmatory studies.

## Materials and Methods

### Data Source

2009 rural-to-urban migrants survey in China project (RUMiC) was used here. RUMiC was initiated by a group of researchers at the Australian National University, the University of Queensland and the Beijing Normal University, and was supported by the Institute for the Study of Labor (IZA). The content validity of the measure was assessed by the RUMiC expert panel. All items were agreed upon so that they were relevant to the context of rural population in China. Till now, there has been no rural survey dataset in China covered psychological measurements more than RUMiC. Additionally, RUMiC included GHQ-12 for rural population in China. Thus, when it comes to mental health of rural residents in China, academic circles have no alternative but RUMiC. The survey was conducted by Datasea Marketing Research, a survey organization. The survey covered 15 cities in nine provinces or metropolitan areas.

### Instruments

The GHQ-12 item scores were coded according to the Likert method (all items coded 0-1-2-3). The responses to positive statements were 3 = more so than usual or better than usual, 2 = same as usual, 1 = less so than usual or less than usual, and 0 = much less than usual. The responses to negative psychological feelings were 0 = not at all, 1 = no more than usual, 2 = rather more than usual, and 3 = much more than usual. In order to explore the factor structure, each item on the GHQ-12 referred to a symptom and was dichotomized into the two values: 0 = “absence of the symptom” and 1 = “presence of the symptom.”

Here, number of kins referred to number of siblings, age ranking, and number of children. Number of siblings denoted the question: “How many siblings do you have (including biological, step and adopted siblings)?” Its response options were 0, 1, 2, 3, 4, 5, 6, and 7 or above. Age ranking denoted the question: “What is your age ranking among your siblings?”. Its response options were 1, 2, 3, 4, 5, 6, and 7 or above. And, number of children denoted the question: “How many children have you ever had?” Its response options were 0, 1, 2, 3, 4, 5, and 6 or above.

### Sample

See Table 1. The mean age of the respondents was 37.03 years old (*SD* = 19.21) ranging from 16 to 126 years in the 2009 sample. More than half of the sample was male, married, employed, and Han ethnicity. There were significant gender differences between marital status, number of sibling, age ranking, and number of children.

**Table 1:** Socio-economic characteristics of the sample.

	Male (*n* = 16,554)	Female (*n* = 15,527)	Chi square	*P* value	Significance
Age	36.58 ± 19.23	37.50 ± 19.18			
Marital status, %			223.7209	0.000	^∗∗∗^
Married	30.54	30.30			
Remarried	1.06	1.22			
Cohabited	0.27	0.26			
Divorced	0.28	0.10			
Widowed	0.98	1.81			
Never married	18.61	14.59			
Ethnicity, %			0.8226	0.364	
Han ethnicity	51.19	47.61			
Ethnic minority	0.60	0.61			
Number of sibling, %			38.2358	0.000	^∗∗∗^
0	2.78	2.22			
1	9.40	8.05			
2	14.69	14.00			
3	10.62	10.68			
4	6.81	6.66			
5	3.89	3.61			
6	2.21	1.85			
7 or above	1.37	1.14			
Age ranking, %			19.1676	0.004	^∗∗∗^
1	20.52	19.88			
2	16.73	15.59			
3	7.91	7.46			
4	3.81	3.13			
5	1.64	1.31			
6	0.66	0.65			
7 or above	0.39	0.32			
Number of children, %			14.8834	0.021	^∗∗^
0	1.42	1.25			
1	16.47	16.31			
2	18.38	19.03			
3	8.02	8.58			
4	3.11	3.38			
5	1.02	1.23			
6 or above	0.80	1.01			

*^∗∗^*p* < 0.05, ^∗∗∗^*p* < 0.01.*

### Statistical Procedure

There were three stages of the analysis. In the first stage, a measuring method of mental disorder was presented. If Mean GHQ1 + Mean GHQ2 + … + Mean GHQ12 > 1, the mental health of the migrants could be considered as poor status. In the second stage, principal component analysis (PCA) with varimax rotation was employed to yield factor structures. The indexes testing sample sphericity were KMO adequacy, Bartlett’s test, and eigenvalues. Then, exploratory factor analysis (EFA) was used to identify the potential factor structure of the GHQ-12. EFA was conducted using SPSS version 24.0 computer software. The final stage was to compute and explain confirmatory factor analysis (CFA) with structural equation model (SEM). Coefficients based on SEM were proposed to improve the routine reporting of psychometric internal consistency (Bentler, 2009). Hence, reliability was measured by Cronbach’s alpha and reflected by goodness of fit of CFA. On the basis of EFA, SEM was performed in order to reflect the CFA model. The seven indexes were used to measure goodness-of-fit and assess model adequacy: root mean squared error of approximation (RMSEA), Akaike’s information criterion (AIC), Bayesian information criterion (BIC), comparative fit index (CFI), Tucker-Lewis index (TLI), standardized root mean squared residual (SRMR), and coefficient of determination (CD). If Cronbach’s alpha score of a factor was more than 0.7, internal consistency of the factor was considered to be satisfactory. CFAs were completed using Stata 14.0 (Stata Corporation, TX, United States).

## Results

### Mental Health Status

In Table 2, mental health status within the numbers of kins was presented. Seemly, number of kins was associated with mental health among the rural residents. There were eleven scenarios with Σmean GHQi > 1. Likewise, the maximal value of Σmean GHQi was 2.317. Thus, minor psychiatric disorders were prevalent in the part of the sample.

**Table 2:** Mental health status within the numbers of kins.

	Σmean GHQi	Judgment	Status
Number of sibling = 0	1.187	>1	Poor
Number of sibling = 1	0.968	<1	Good
Number of sibling = 2	0.889	<1	Good
Number of sibling = 3	0.984	<1	Good
Number of sibling = 4	0.994	<1	Good
Number of sibling = 5	1.004	>1	Poor
Number of sibling = 6	1.048	>1	Poor
Number of sibling ≥ 7	1.023	>1	Poor
Age ranking = 1	1.006	>1	Poor
Age ranking = 2	0.948	<1	Good
Age ranking = 3	0.939	<1	Good
Age ranking = 4	1.018	>1	Poor
Age ranking = 5	0.867	<1	Good
Age ranking = 6	1.038	>1	Poor
Age ranking ≥ 7	0.759	<1	Good
Number of children = 0	0.993	<1	Good
Number of children = 1	0.719	<1	Good
Number of children = 2	0.850	<1	Good
Number of children = 3	1.262	>1	Poor
Number of children = 4	1.666	>1	Poor
Number of children = 5	2.027	>1	Poor
Number of children ≥ 6	2.317	>1	Poor

### EFA Analysis

Principal component analysis with varimax rotation solution of the various models could be seen in Table 3. Each rotated factor was considered to be composed of subtests with loadings bigger than 0.30. In the sample, KMO statistic indicated adequate sample size for the factor analysis. Bartlett’s test of sphericity was significant. There were two or three factors were extracted in the sample since the eigenvalues were more than 1.

**Table 3:** Factor analysis of GHQ-12 regarding the number of siblings, age ranking, and number of children.

	KMO adequacy	Bartlett’s sphericity	First eigenvalue	Second eigenvalue	Final eigenvalue	Component number	Cumulative %
Number of sibling = 0	0.882	1.915E3	5.413		1.080	2	54.109
Number of sibling = 1	0.906	6.609E3	5.099		1.146	2	52.044
Number of sibling = 2	0.891	1.038E4	4.507	1.168	1.006	3	55.670
Number of sibling = 3	0.897	1.065E4	4.640		1.181	2	48.510
Number of sibling = 4	0.903	8.986E3	4.866		1.126	2	49.932
Number of sibling = 5	0.899	5.160E3	4.732		1.110	2	48.679
Number of sibling = 6	0.893	3.471E3	5.029		1.132	2	51.346
Number of sibling ≥ 7	0.873	1.875E3	4.640	1.206	1.103	3	57.909
Age ranking = 1	0.906	1.852E4	4.917		1.117	2	50.282
Age ranking = 2	0.897	1.351E4	4.630		1.211	2	48.671
Age ranking = 3	0.891	7.364E3	4.493		1.110	2	46.694
Age ranking = 4	0.905	4.344E3	4.863		1.060	2	49.363
Age ranking = 5	0.869	1.810E3	4.530	1.260	1.028	3	56.824
Age ranking = 6	0.854	1.306E3	5.248	1.320	1.090	3	63.812
Age ranking ≥ 7	0.741	767.868	5.040	1.327	1.065	3	61.942
Number of children = 0	0.803	831.866	5.282	1.421	1.040	3	64.529
Number of children = 1	0.888	1.135E4	4.441		1.222	2	47.191
Number of children = 2	0.880	1.616E4	4.381	1.181	1.009	3	54.767
Number of children = 3	0.897	8.366E3	4.747		1.127	2	48.952
Number of children = 4	0.921	4.153E3	5.361		1.089	2	53.747
Number of children = 5	0.911	1.567E3	5.666		1.305	2	58.087
Number of children ≥ 6	0.873	872.037	5.748	1.332	1.048	3	67.741

*Bartlett’s sphericity was assessed by Approx. Chi-Square.*

### CFA Analysis

In Table 4, CFA revealed that the two, and three factors gave the best goodness-of-fit among the various models for factor structures. The minimum size of component consisted of two GHQ items, while the maximal size of component consisted of ten GHQ items. Cronbach’s alpha of the various models could be seen in Table 5. The minimum value of Cronbach’s alpha was 0.3887, while the maximal value of Cronbach’s alpha was 0.8751.

**Table 4:** The item construction of the main components.

	Component 1	Component 2	Component 3
Number of sibling = 0	GHQ1 GHQ3 GHQ4	GHQ2 GHQ5 GHQ6 GHQ7 GHQ8 GHQ9 GHQ10 GHQ11 GHQ12	
Number of sibling = 1	GHQ3 GHQ4	GHQ1 GHQ2 GHQ5 GHQ6 GHQ7 GHQ8 GHQ9 GHQ10 GHQ11 GHQ12	
Number of sibling = 2	GHQ3 GHQ4	GHQ1 GHQ2 GHQ5 GHQ6 GHQ8 GHQ9 GHQ10 GHQ11	GHQ7 GHQ12
Number of sibling = 3	GHQ1 GHQ3 GHQ4	GHQ2 GHQ5 GHQ6 GHQ7 GHQ8 GHQ9 GHQ10 GHQ11 GHQ12	
Number of sibling = 4	GHQ1 GHQ3 GHQ4 GHQ8	GHQ2 GHQ5 GHQ6 GHQ7 GHQ9 GHQ10 GHQ11 GHQ12	
Number of sibling = 5	GHQ3 GHQ4	GHQ1 GHQ2 GHQ5 GHQ6 GHQ7 GHQ8 GHQ9 GHQ10 GHQ11 GHQ12	
Number of sibling = 6	GHQ1 GHQ3	GHQ2 GHQ4 GHQ5 GHQ6 GHQ7 GHQ8 GHQ9 GHQ10 GHQ11 GHQ12	
Number of sibling ≥ 7	GHQ3 GHQ4 GHQ5	GHQ2 GHQ6 GHQ7 GHQ8 GHQ9 GHQ10 GHQ11	GHQ1 GHQ12
Age ranking = 1	GHQ1 GHQ3 GHQ4	GHQ2 GHQ5 GHQ6 GHQ7 GHQ8 GHQ9 GHQ10 GHQ11 GHQ12	
Age ranking = 2	GHQ3 GHQ4	GHQ1 GHQ2 GHQ5 GHQ6 GHQ7 GHQ8 GHQ9 GHQ10 GHQ11 GHQ12	
Age ranking = 3	GHQ1 GHQ3 GHQ4	GHQ2 GHQ5 GHQ6 GHQ7 GHQ8 GHQ9 GHQ10 GHQ11 GHQ12	
Age ranking = 4	GHQ1 GHQ3 GHQ4	GHQ2 GHQ5 GHQ6 GHQ7 GHQ8 GHQ9 GHQ10 GHQ11 GHQ12	
Age ranking = 5	GHQ1 GHQ3 GHQ10 GHQ11	GHQ4 GHQ5 GHQ6 GHQ8 GHQ9	GHQ2 GHQ7 GHQ12
Age ranking = 6	GHQ1 GHQ3 GHQ4	GHQ2 GHQ6 GHQ7 GHQ8 GHQ9 GHQ10 GHQ11	GHQ5 GHQ12
Age ranking ≥ 7	GHQ1 GHQ4 GHQ10 GHQ11	GHQ2 GHQ3 GHQ5 GHQ8 GHQ9	GHQ6 GHQ7 GHQ12
Number of children = 0	GHQ6 GHQ8 GHQ12	GHQ1 GHQ2 GHQ5 GHQ7 GHQ9 GHQ10 GHQ11	GHQ3 GHQ4
Number of children = 1	GHQ3 GHQ4	GHQ1 GHQ2 GHQ5 GHQ6 GHQ7 GHQ9 GHQ10 GHQ11 GHQ12	
Number of children = 2	GHQ3 GHQ4	GHQ1 GHQ2 GHQ5 GHQ6 GHQ8 GHQ9 GHQ10 GHQ11	GHQ7 GHQ12
Number of children = 3	GHQ1 GHQ3 GHQ4 GHQ8	GHQ2 GHQ5 GHQ6 GHQ7 GHQ9 GHQ10 GHQ11 GHQ12	
Number of children = 4	GHQ1 GHQ3 GHQ4	GHQ2 GHQ5 GHQ6 GHQ7 GHQ8 GHQ9 GHQ10 GHQ11 GHQ12	
Number of children = 5	GHQ1 GHQ3 GHQ4	GHQ2 GHQ5 GHQ6 GHQ7 GHQ8 GHQ9 GHQ10 GHQ11 GHQ12	
Number of children ≥ 6	GHQ5 GHQ6 GHQ9 GHQ10	GHQ1 GHQ2 GHQ7 GHQ8 GHQ11	GHQ3 GHQ4 GHQ12

**Table 5:** Cronbach’s alpha regarding number of siblings, age rank, and number of children.

	Component 1	Component 2	Component 3	Assessment
Number of sibling = 0	0.6531	0.8685		Acceptable
Number of sibling = 1	0.6986	0.8473		Acceptable
Number of sibling = 2	0.6686	0.7838	0.6439	Acceptable
Number of sibling = 3	0.6875	0.8118		Acceptable
Number of sibling = 4	0.7116	0.8292		Acceptable
Number of sibling = 5	0.6428	0.8200		Acceptable
Number of sibling = 6	0.5908	0.8471		Acceptable
Number of sibling ≥ 7	0.6546	0.7714	0.3887	Acceptable
Age ranking = 1	0.7004	0.8307		Acceptable
Age ranking = 2	0.6842	0.8214		Acceptable
Age ranking = 3	0.6441	0.8057		Acceptable
Age ranking = 4	0.6646	0.8286		Acceptable
Age ranking = 5	0.5523	0.7195	0.5087	Satisfactory
Age ranking = 6	0.6475	0.8390	0.6992	Acceptable
Age ranking ≥ 7	0.5613	0.7726	0.4052	Acceptable
Number of children = 0	0.4828	0.8462	0.7128	Acceptable
Number of children = 1	0.6349	0.8007		Acceptable
Number of children = 2	0.6382	0.7785	0.6224	Acceptable
Number of children = 3	0.7129	0.8150		Acceptable
Number of children = 4	0.7343	0.8541		Acceptable
Number of children = 5	0.7908	0.8751		Acceptable
Number of children ≥ 6	0.8328	0.7165	0.6302	Acceptable

See Table 6. Not all fit indices of the models had satisfactory goodness of fit. Based on Cronbach’s alpha statistic and CFA Goodness of Fit, CFA model with two siblings was superior to the other CFA models with and without siblings. CFA models with age ranking 1 and 4 were superior to the other CFA models with age ranking. CFA models with 3 and 4 children were superior to the other CFA models with children.

**Table 6:** Good-for-fit indexes for CFA models of GHQ-12.

	RMSEA	AIC	BIC	CFI	TLI	SRMR	CD	Obs.
Number of sibling = 0	0.112	–115.613	29.585	0.868	0.835	0.060	0.955	1,556
Number of sibling = 1	0.091	–3657.866	–3459.051	0.893	0.867	0.049	0.944	5,438
Number of sibling = 2	0.071	–8367.769	–8131.076	0.921	0.897	0.042	0.965	8,934
Number of sibling = 3	0.082	–5334.897	–5111.364	0.896	0.871	0.048	0.931	6,627
Number of sibling = 4	0.078	–3837.620	–3623.981	0.915	0.894	0.039	0.938	4,192
Number of sibling = 5	0.094	–2190.088	–1984.356	0.872	0.834	0.051	0.865	2,333
Number of sibling = 6	0.106	–1000.363	–826.209	0.858	0.823	0.058	0.932	1,263
Number of sibling ≥ 7	0.105	–133.352	45.021	0.859	0.818	0.058	0.903	782
Age ranking = 1	0.081	–8301.603	–8061.862	0.909	0.887	0.044	0.941	12,072
Age ranking = 2	0.085	–7931.205	–7698.931	0.888	0.860	0.049	0.934	9,665
Age ranking = 3	0.082	–4199.477	–3987.089	0.888	0.861	0.047	0.917	4,591
Age ranking = 4	0.081	–1363.183	–1175.723	0.906	0.883	0.044	0.926	2,077
Age ranking = 5	0.104	–1610.371	–1445.000	0.839	0.791	0.068	0.919	879
Age ranking = 6	0.134	–366.581	–231.327	0.829	0.778	0.078	0.984	390
Age ranking ≥ 7	0.106	–373.336	–198.554	0.876	0.839	0.052	0.885	211
Number of children = 0	0.169	–354.682	–240.238	0.748	0.673	0.082	0.983	555
Number of children = 1	0.088	–18239.461	–18029.245	0.883	0.850	0.048	0.918	6,780
Number of children = 2	0.080	–15011.945	–14756.934	0.896	0.866	0.046	0.960	7,738
Number of children = 3	0.076	1837.147	2049.936	0.914	0.892	0.040	0.936	3,432
Number of children = 4	0.078	3048.422	3227.045	0.927	0.909	0.043	0.948	1,343
Number of children = 5	0.087	1476.421	1613.337	0.922	0.903	0.054	0.965	464
Number of children ≥ 6	0.102	–2704.619	–2504.483	0.881	0.847	0.054	0.924	374

## Discussion

Using the 2009 RUMiC, this study explored the factor structures of GHQ-12 within the various numbers of kins. The construct validity of the constructs underlying the 12 items was conducted by a principal component factor analysis with varimax rotation. In the present paper, two-factor and three-factor solution were confirmed to be accepted after EFA and CFA analysis. The internal consistency was assessed by Cronbach’s alpha and CFA, part of which showed satisfactory results among the rural residents with two siblings, age ranked 1 and 4, and 3 and 4 Children. This suggested numbers of kins could associate with the mental health of the rural residents.

Consistent with previous studies in other countries, the reliability and validity of CHQ-12 were moderately acceptable. Reliability analysis of RUMiC version of the GHQ-12 showed satisfactory result (Cronbach’s alpha coefficient > 0.75). According to the result from Quek et al. (2001), each of the 12 items with Cronbach’s alpha value with 0.37–0.79 was acceptable. To date, the lowest value of Cronbach’s alpha of GHQ-12 provided by Bakhla et al. (2013) was 0.7. Their factors with Cronbach’s alpha coefficients were 0.70, 0.59, and 0.34, respectively. Thus, the present Cronbach’s alpha values of most items were higher in this study. Compared with one-factor model (e.g., Fernandes and Vasconcelos-Raposo, 2013; Romppel et al., 2013), two-factor model (e.g., Jacob et al., 1997; Kalliath et al., 2004; Rahmati Najarkolaei et al., 2014), and three-factor model (e.g., Kuruvilla et al., 1999; Daradkeh et al., 2001; Cheung, 2002; Padrón et al., 2012), this study presented much more scientific rigors regarding co-existing two-factor, and three-factor structures of the GHQ-12. But, factor structures of GHQ-12 within the number of kins might be influenced by translation biases (Pan and Goldberg, 1990), wording effects (Ye, 2009; Wang and Lin, 2011; Abubakar and Fischer, 2012; Molina et al., 2014), cultural biases (Lewis and Araya, 1995), interviewing biases (Gao et al., 2012), and clinical biases (Le Fevre et al., 1999).

The results presented in this study were in line with current status in rural China that numbers of kins played an important role in family care. For example, for rural older adults living only with children, their mental health was highly contingent on their family ties (Tang et al., 2019). Another study also showed having no family caregivers is important factors associated with a worse treatment status of people with severe mental illness in contemporary rural China (Ran et al., 2019). Thus, several scholars confirmed the importance and need to increase the public’s awareness and knowledge about common mental disorders (Yu et al., 2016).

This study also gave insights into discussion of prevention and treatment of mental disorders in rural China. A cross-sectional study verified the strongly negative effect of the mental health dimension on subjective well-being in rural China (Wang et al., 2015). Thus, several medical scientists appealed to local government to promote mental illness recognition within rural areas (Huang et al., 2019). Seemingly, the finding of this study supported current reform of child policy in China. In China, one-child policy contributed to the general deteriorating trend in physical and mental health of the bereaved parents’ life after their only child’s death (Zheng et al., 2017). Although two-child policy had also caused medical problems faced by older second-child pregnant women (Li and Deng, 2017; Fu et al., 2018), this study reported mental health with one-child, and two-child were good. Likewise, multiple children policy should be postponed because it could worsen the mental health among the rural residents.

The limitations and future studies should be highlighted. First, factor structures of GHQ-12 might depend on number of kins among the rural residents with cross-sectional data. Future study can analyze them for the general population with longitudinal data. Second, the main findings of this study sourced from the sample background dominated by middle-aged, married, and employed male with Han ethnicity. Future study could adopt the sample from the single, the unemployed, females, and the older or ethnic minority. Furthermore, future studies of mental health service utilization in rural populations should be given special attention to family characteristics considering structure, relationship, and number.

## Conclusion

The findings from the present study revealed the mental health of rural residents measured by GHQ-12 within the number of kins in rural China. The finding of satisfactory factor structures and psychometric properties for the RUMiC version of the GHQ-12 in this study also supported the decision to screen and identify mental health of rural populations within the specific number of kins. The findings could inform the design of prevention programs of mental illness that targets Chinese rural residents.

## Data Availability

All datasets generated for this study are included in the manuscript and/or the supplementary files.

## Author Contributions

MG designed the study, performed the statistical analysis, and completed the original version of the manuscript. BH redesigned, read, polished, revised, and approved the final version of the manuscript.

## Conflict of Interest Statement

The authors declare that the research was conducted in the absence of any commercial or financial relationships that could be construed as a potential conflict of interest.
